# Development and psychometric evaluation of a context-based parental self-efficacy instrument for healthy dietary and physical activity behaviors in preschool children

**DOI:** 10.1186/s12966-016-0438-y

**Published:** 2016-10-20

**Authors:** Benjamin Bohman, Finn Rasmussen, Ata Ghaderi

**Affiliations:** 1 Department of Clinical Neuroscience, Karolinska Institutet, Stockholm, Sweden; 2 Centre for Psychiatric Research, Stockholm Health Care Services, Stockholm County Council, Liljeholmstorget 7 B, SE-117 63 Stockholm, Sweden; 3 Department of Public Health Sciences, Karolinska Institutet, Stockholm, Sweden; 4 Centre for Epidemiology and Community Medicine, Stockholm Health Care Services, Stockholm County Council, Stockholm, Sweden

**Keywords:** Diet, Pediatric obesity, Physical activity, Self-efficacy

## Abstract

**Background:**

Parental self-efficacy (PSE) refers to beliefs of parents to effectively engage in behaviors that result in desired outcomes for their children. There are several instruments of PSE for promoting healthy dietary or physical activity (PA) behaviors in children. These measures typically assess PSE in relation to some quantity or frequency of behavior, for example, number of servings or times per week. However, measuring PSE in relation to contextual circumstances, for example, psychological states and situational demands, may be a more informative approach. The purpose of the present study was to develop and psychometrically evaluate a context-based PSE instrument.

**Methods:**

Swedish mothers of five-year-old children (*n* = 698) responded to the Parental Self-Efficacy for Healthy Dietary and Physical Activity Behaviors in Preschoolers Scale (PDAP) and a questionnaire on dietary and PA behaviors in children. Interviews were conducted to explore participant perceptions of the quality of the PDAP items. Psychometric evaluation was conducted using exploratory and confirmatory factor analyses. Spearman correlations between PSE and child behaviors were examined.

**Results:**

Twenty-seven interviews were conducted with participants, who perceived the items as highly comprehensible, relevant and acceptable. A four-factor model of a revised 21-item version of the PDAP fitted the data, with different factors of PSE for promoting healthy dietary or PA behaviors in children depending on whether circumstances were facilitating or impeding successful performance. Internal consistency was excellent for total scale (Cronbach’s α = .94), and good for factors (α = .84–.88). Correlations were in the expected direction: positive correlations between PSE and healthy behaviors, and negative correlations between PSE and unhealthy behaviors (all *r*_s_s ≤ .32).

**Conclusions:**

Psychometric evaluation of the PDAP provided preliminary support of construct validity and internal consistency.

## Background

Childhood obesity is a major public health concern worldwide. In 2013, it was estimated that the global prevalence of overweight and obesity in children under the age of five reached over 42 million [1]. Obesity in childhood is associated with a range of adverse health effects in the short-term, including atherosclerosis, type 2 diabetes mellitus, and musculoskeletal disorders [2]. Furthermore, childhood obesity is associated with low self-esteem and quality of life [3]. Obese children run an increased risk of becoming obese adults [4], and childhood obesity is associated with long-term morbidity and premature mortality in adulthood [5]. Efficacious prevention and treatment of childhood obesity includes a combination of dietary, physical activity (PA), and behavior change interventions within a family-oriented approach [6, 7].

In a review of behavior change theories in obesity prevention interventions in children aged 4 to 6 years, the most commonly used framework was social cognitive theory (SCT) [8]. Indeed, successful interventions were more likely to use behavioral change strategies consistent with SCT, including strategies to increase self-efficacy (SE) in parents. Perceived SE is the core construct in SCT and refers to beliefs in one’s capabilities to reach desired goals through one’s actions [9], with parental self-efficacy (PSE) referring to parents’ beliefs in their capabilities to engage in effective behavior to reach desired goals for their children. An example of PSE is a mother’s beliefs in her capabilities to promote healthy dietary behaviors in her child. SCT is based on an agentic perspective on human development, adaptation, and change, which means that individuals are intentionally influencing their functioning and life conditions [10]. According to SCT, SE is the central mechanism of human agency, and thus the foundation of human motivation and action [9, 11]. People would not engage in behaviors if they did not believe that their actions could produce desired effects, and they would not persevere in the face of difficulties. Efficacy beliefs are context-dependent: people do not demonstrate a general sense of personal efficacy expanding all spheres of functioning, rather, SE is specific to a certain domain, and thus varies across domains [12]. This is referred to as domain-specificity [9]. For example, an individual may have high SE for engaging in healthy dietary behaviors, but low SE for managing household economy. However, efficacy beliefs may be associated across proximate domains, for example, for promoting dietary and PA behaviors in children; behaviors that tend to cluster [13]. This is referred to as between-domain generality [9]. Also within a single domain, the level of strength of efficacy beliefs may vary depending on contextual factors, for example, psychological states, time constraints, and task demands [9]. Thus, SE is not a global disposition or general trait that manifest uniformly irrespective of context [9, 12]. On the contrary, in promoting healthy PA behaviors in children, for example, personal circumstances such as perceived stress, or situational circumstances such as limited availability of PA settings, may be experienced by parents as hampering their performance, which in turn may result in lowered PSE. Consistent with their status as a mechanism of behavior change in SCT, efficacy beliefs have been demonstrated to mediate change across a range of behavioral domains [9]. However, there is a lack of mediation studies of PSE in the domains of dietary and PA behaviors in children. This is unfortunate since in childhood obesity interventions PSE may be a particularly important construct, as dietary and PA behaviors in children to a large extent are under the control of parents [14].

Valid instruments provide differential information on efficacy beliefs by including a range of behaviors, and personal and situational circumstances that may impact on successful performance [15–17]. There are several instruments available of PSE for dietary and PA behaviors in children. However, a majority of them suffers from conceptual or methodological limitations. These include a restricted number of items, which may not provide sufficient coverage of a domain or allow PSE to vary in belief strength due to contextual factors (e.g., [18]), and a response format with only a few categories, for example, “a 4-point Likert response, where 1 = not at all confident and 4 = very confident” [19], rendering assessment of different levels of strength difficult. Some instruments consist of non-specific items, for example, “change your family’s eating patterns to keep your child from being overweight” [20], which may limit the possibility to detect changes in PSE following intervention. Other instruments may be less appropriate for evaluating processes of change in childhood obesity interventions targeting both dietary and PA behaviors as these measures assess only a single behavior, for example, PA (e.g., [21]), or a limited set of behaviors, for example, fruit, juice, and vegetable consumption (e.g., [22]). In addition, few measures have been evaluated psychometrically using factor analysis on larger samples (e.g., [23]), if at all (e.g., [24]).

PSE instruments concerning dietary and PA behaviors in children typically assess PSE in relation to some quantity or frequency of behavior, often with reference to national health guidelines. An item example is: “How confident are you that your child plays outside or is active in sports for a total of at least 60 min on most days of the week?” [25]. However, basing PSE items on quantity or frequency without taking contextual circumstances into account (e.g., [18, 20, 25]) may not be the optimal approach. An example of a context-based PSE item is: “Rate your confidence to influence your child’s physical activity when the child is not interested” [26]. There are at least two reasons to why context may be more informative than quantity or frequency. First, assessment of efficacy beliefs runs the risk of becoming too hypothetic or even speculative if the individual has limited or no previous experience of the target behavior, for example, daily consumption of five servings of fruits and vegetables or to use vegetable oils when cooking. Second, obesity-related behaviors do not occur in isolation, rather, they are dependent on contextual factors [27], and, as stated above, so are efficacy beliefs. In addition, an advantage of focusing on context is that it may provide better indications of what behaviors to target and when in childhood obesity interventions.

The purpose of the present study was to develop and provide psychometric evidence for a PSE instrument for healthy dietary and PA behaviors in preschool children. In contrast to previous PSE measures, the items were designed exclusively to reflect contextual factors that either facilitate or impede PSE for promoting healthy dietary and PA behaviors in children. We hypothesized that: (a) participating mothers would perceive the items as comprehensive, relevant, and acceptable, (b) confirmatory factor analysis (CFA) of a model of context-based PSE would fit the data better than two rival non-context-based models, and (c) the PSE items would be positively correlated with healthy parent-reported dietary and PA behaviors in children and negatively correlated with unhealthy child behaviors.

## Methods

### Sample selection

From the population of Swedish first-time mothers of five-year-old children who had their children from July to September in 2006 when 22 to 36 years of age (*n* = 9560, according to the Statistics Sweden Register of the Total Population), a sample of 3500 mothers was randomly selected in October 2011 as potential participants in the study. Since PSE may be different in females and males [28] and collecting data on both mothers and fathers and conducting gender-specific analyses was considered too resource demanding and not critical for the purpose of the present study, only mothers were chosen as participants.

### Measurement

#### Parental self-efficacy

The Parental Self-Efficacy for Healthy Dietary and Physical Activity Behaviors in Preschoolers Scale (PDAP) was developed by the authors, who created items based on (a) previous personal experience of PSE scale development and evaluation in the domains of dietary and PA behaviors in children [29, 30], (b) SE literature (e.g., [9, 15]), and (c) existing PSE measures (for examples, see the Background). The PDAP consists of 24 items covering contextual circumstances that may either facilitate or impede PSE for promoting healthy dietary and PA behaviors in preschool children (see Table 1). Items cover parental behaviors which research has identified as important for promotion of healthy behaviors in these domains [14]. Examples of parental behaviors in the dietary domain include creating a positive atmosphere when having meals and being a role model for healthy eating and drinking. Items also cover psychological states of parents and children and situational circumstances that may impair successful performance. Items are distributed onto two subscales, one for dietary behaviors, and one for PA behaviors. Definitions of PSE, healthy and unhealthy foods and beverages, and PA and sedentary behaviors precede the items. Responses are made on an 11-point Likert scale, with anchors at 0 (*not at all confident*), 5 (*moderately confident*), and 10 (*completely confident*). Participants were instructed to respond to the items with their 5-year-old child in mind.

**Table 1:** Means, SDs, and Factor Loadings for Exploratory Factor Analysis using the Minimum Residuals procedure after Promax Rotation on Parental Self-Efficacy Items (*n* = 349)

Parental self-efficacy items	Mean (*SD*)^a^	Factor loadings			
		Factor 1^b^	Factor 2	Factor 3	Factor 4
How confident are you that you can…					
1. Prioritize spending money on purchasing healthy foods and beverages, instead on purchasing foods and beverages high in saturated fat or sugar?	8.59 (1.42)	.**96**	-.08	.02	-.04
2. Prioritize spending time on locating healthy foods and beverages for purchase when such products are not immediately available?	7.30 (1.87)	.**66**	.05	-.08	.12
3. Prepare and serve healthy foods and beverages in an appetizing way?	8.39 (1.42)	.**67**	.16	.11	-.05
4. Create a positive atmosphere when having meals with healthy choices?	8.21 (1.42)	.**61**	.11	.14	.01
5. Be a role model for your child about healthy eating and drinking?	8.51 (1.45)	.**65**	.09	.15	-.11
How confident are you that you can get your child to eat healthy foods and drink healthy beverages…					
6. When you yourself want to consume foods and beverages that are not healthy?	5.95 (2.53)	.07	.13	-.19	.56
7. When your child wants to consume foods and beverages that are not healthy?	7.81 (1.52)	-.03	.**75**	.03	.07
8. When you are tired, stressed, emotionally upset, or affected by daily hassles?	6.52 (1.85)	.26	.**60**	-.16	.11
9. When your child is acting defiant?	7.16 (1.84)	.07	.**76**	-.03	.07
10. When eating out?	5.53 (1.99)	.25	.20	-.12	.38
11. When your child is having a friend over?	7.50 (1.71)	.08	.**62**	.17	-.01
12. During holidays, on vacation, or in similar situations?	6.64 (1.81)	.23	.29	.09	.22
How confident are you that you can…					
13. Prioritize spending money on purchasing toys, equipment, or the like, for use in physical activity, for example, a trampoline or skates?	8.70 (1.57)	.22	-.11	.**45**	.20
14. Prioritize spending time on taking your child outdoors for physical activity, for example, to a playground or for cycling?	8.65 (1.43)	.04	-.11	.**80**	.16
15. Arrange opportunities for your child to engage in different kinds of physical play or activities which he or she finds amusing?	8.68 (1.37)	.06	-.11	.**84**	.08
16. Create a positive atmosphere when being physically active with your child?	8.71 (1.34)	.12	.12	.**77**	-.09
17. Be a role model for your child about a physically active lifestyle, for example, by engaging in physical play, taking walks, or doing sports?	8.38 (1.65)	.16	.00	.**62**	.04
How confident are you that you can get your child to be physically active…					
18. When you yourself want to be sedentary?	6.47 (2.10)	-.13	-.05	.16	.**77**
19. When your child wants to be sedentary, for example, play computer games or watch TV?	7.09 (1.71)	-.04	.16	.16	.**63**
20. When you are tired, stressed, emotionally upset, or affected by daily hassles?	6.26 (1.86)	-.03	.05	.10	.**76**
21. When your child is acting defiant?	6.23 (2.04)	.02	.06	.08	.**70**
22. Outdoors, when the weather is bad, for example, when it is rainy or cold?	6.52 (2.16)	.07	-.13	.29	.**53**
23. When your child is having a friend over?	7.59 (1.70)	-.18	.27	.**56**	.15
24. During holidays, on vacation, or in similar situations?	8.48 (1.55)	-.12	.37	.**60**	-.01

Bold numbers indicate on what factor items loaded the highest. Items 6, 10, and 12 were excluded from the factor solution

^a^Item response alternatives range from 0 to 10

^b^Factor labels. Contextual circumstances that 1) Facilitate parental self-efficacy (PSE) for promoting healthy dietary behaviors in children; 2) Impede PSE for promoting healthy dietary behaviors in children; 3) Facilitate PSE for promoting healthy PA behaviors in children; and 4) Impede PSE for promoting healthy PA behaviors in children

To explore participant perceptions of the quality of the PDAP items, participants were invited to take part in semi-structured interviews. For each item, the interviewers requested participants to indicate the extent to which it was comprehensible, relevant, and acceptable (i.e., non-offending) on Likert scales ranging from 0 (*difficult to comprehend/not relevant/not acceptable*) to 5 (*easy to comprehend/very relevant/completely acceptable*). If respondents indicated 2 or less, interviewers asked follow-up questions to inquire about the reasons for the low score.

#### Dietary and physical activity behaviors in children

Correlations between PSE and parent-reported dietary and PA behaviors in children were assessed using a food frequency questionnaire (FFQ) and questions on PA and sedentary behaviors (for items, see Table 3). The FFQ has previously been validated against an eight-day food diary completed during two four-day records in a childhood obesity prevention trial, showing correlations in the medium to large range [31]. Dietary items covered frequency of dietary behaviors when not at day care on 13- or 17-point response scales, for example, number of occasions of vegetable intake per month (zero to three occasions), or per week (one to six occasions), or per day (one to six or more occasions). Items to assess number of hours of PA and sedentary behaviors when not at day care were developed as part of the study and measured on a 14-point response scale ranging from zero to six hours or more, with a 0.5 h interval between points.

### Data collection procedure

A letter was sent by mail to the randomly selected sample of 3500 mothers, informing them about the study and inviting them to participate in a web-based survey. The survey included demographic and anthropometric questions, the PDAP, and items about dietary and PA behaviors in children. Responding to all survey items was made mandatory for participation; thus there were no missing values. In the survey, mothers were invited to participate in interviews by telephone about the quality of the PDAP items. Participants were interviewed consecutively, starting with the first mother who agreed to be interviewed. Interviews continued until it was determined that no additional information emerged concerning the quality of the PDAP items (i.e., saturation). Birth date of mothers and children, and information on mothers’ marital status were requested from the Swedish Tax Agency. Implied consent was indicated by mothers responding to the survey. The study was approved by the regional ethical review board in Stockholm, Sweden (2011/1325–31).

### Data analysis

Data analyses were performed using the SPSS (Version 21, SPSS Inc., Chicago, IL) and the LISREL (Version 9.10, SSI, Inc., Skokie, IL). The sample of 698 participants was randomly split into two subsamples. Exploratory factor analysis (EFA) was performed on one subsample (*n* = 349) and CFAs on the other subsample (*n* = 349). As data were ordinal and there were indications of multivariate non-normality, EFA was performed using Minimum Residuals (MINRES), which is a procedure based on direct minimization of least squares. MINRES does not require any distributional assumptions and can be used with matrices of polychoric correlations [32]. In the EFA, oblique (Promax) rotation was used. Internal consistency for the PDAP and its factors was assessed using Cronbach’s α, which was interpreted as α ≥ .70 = acceptable, α ≥ .80 = good, and α ≥ .90 = excellent. Three models of the PDAP were tested using CFA: a four-factor model of context-based PSE with factors obtained using EFA, a two-factor model based on the dietary and PA behaviors subscales (items 1–12 and 13–24, respectively), and a one-factor model with PSE as the latent variable. The one- and two-factor models were tested as rival alternatives to the context-based model. Although each item of the PDAP is context-based, the one-factor model containing all items does not take into account the fact that items represent different contextual factors, that is, situations facilitating or impeding PSE, across and within domains (i.e., diet, PA). Similarly, the two-factor model takes into account only contextual factors within domains, as it is based on the dietary and PA subscales of the instrument. Due to multivariate non-normality, CFA was performed using maximum likelihood robust estimation. CFA models were modified to improve model fit by allowing correlations of error terms between manifest variables (i.e., PSE items), based on modification indices [33], but only within each factor and only when it theoretically made sense to avoid over-fitting. The following indices were used to evaluate model fit: root mean square error of approximation (RMSEA), comparative fit index (CFI), standardized root mean square residual (SRMR), and parsimony goodness-of-fit index (PGFI). Tabachnick and Fidell [33] provide recommendations for interpreting model fit using these indices. RMSEA values of .06 or less indicate a good-fitting model, whereas values greater than .10 suggest poor-fitting models. CFI values greater than .95 are indicative of good-fitting models. SRMR values of .08 or less are suggestive of good-fitting models. The closer the PGFI value is to 1.00, the better the fit, however, this index will always be substantially smaller than other indices since it is based on the number of estimated parameters relative the number of data points. Correlations between the PDAP and parent-reported dietary and PA behaviors in children were based on the entire sample (*n* = 698) and calculated using Spearman correlation coefficient *r*_s_, as distributions of some of the child behavior variables were non-normal.

## Results

### Participant characteristics

Of the randomly selected sample of 3500 mothers, 698 participated in the study, representing a 20 % response rate. Mean age of participants was 36 years (*SD* = 3, range = 27–43), 426 (61 %) were married, 257 (37 %) were unmarried, 13 (2 %) were divorced, one mother was in a civil partnership, and one had dissolved a civil partnership. Of the participants, three had nine years of schooling (from seven to 15 years of age), 126 (18 %) had an additional three years of schooling at the high school level (from 16 to 18 years of age), 78 (11 %) had studied at a college or university for less than three years, and 470 (68 %) for three years or more, and 21 (3 %) had studied at a PhD program. Self-reported mean body mass index (BMI) was 24 (*SD* = 4, range = 17–40; pregnant women excluded, *n* = 36), and the prevalence of overweight (25 ≤ BMI < 30) and obesity (BMI ≥ 30) was 19 % and 6 %, respectively.

There were differences between participants (*n* = 698) and non-participants (*n* = 2802) concerning age with a mean difference of 1.2 years *t* (1183) = 8.60, *p* < .001 and on marital status (married/in civil partnership, unmarried, divorced/dissolved civil partnership), *χ* ^2^(2) = 18.50, *p* < .001, with a larger proportion of participants being married/in civil partnership (61 %) compared to non-participants (53 %). The groups also differed on ethnicity: 5 % of participants had a country of origin other than Sweden, whereas the corresponding number for non-participants was 14 %, *χ* ^2^(1) = 42.10, *p* < .001.

### Item quality and descriptive analysis

To explore participant perceptions of the quality of the PDAP items, 27 interviews were conducted. Participants perceived items as highly comprehensible, relevant and acceptable. However, items 9 and 21 (see Table 1) contained synonyms to “acting defiant”; these synonyms were removed for the sake of simplicity. Across the 24 PDAP items in the EFA subsample (*n* = 349), means ranged from 5.53 to 8.71 (*SD*s from 1.34 to 2.53), with skewness ranging from −0.26 to–1.47, and kurtosis ranging from 0.04 to 3.63, indicating univariate normality [34]. For item means and *SD*s, see Table 1. Maximum endorsement frequency was 44 % (for the highest response alternative on item 13, meaning that no larger proportion of participants chose a particular response on any other item).

### Exploratory factor analysis and internal consistency

EFA yielded a somewhat complex factor solution, with item 6 loading on an unexpected factor and items 10 and 12 on several factors. These three items were excluded from the factor solution and the subsequent CFA based on the EFA. A four-factor solution emerged of contextual circumstances that: 1) Facilitate PSE for promoting healthy dietary behaviors in children (items 1–5); 2) Impede PSE for promoting healthy dietary behaviors in children (items 7–9 and 11); 3) Facilitate PSE for promoting healthy PA behaviors in children (items 13–17 and 23–24); and 4) Impede PSE for promoting healthy PA behaviors in children (items 18–22). For factor loadings of the PDAP items, see Table 1. In the factor solution, factor correlations ranged from *r* = .50 to *r* = .64. Internal consistency was Cronbach’s α = .94 for the 21 items, α = .86 for Factor 1, α = .84 for Factor 2, α = .88 for Factor 3, and α = .87 for Factor 4. Corrected item-total correlations for the 21 items ranged from *r* = .51 to *r* = .72.

### Confirmatory factor analysis

Three models of the PDAP were hypothesized and tested using CFA: (a) a context-based model with four latent variables based on the EFA (excluding items 6, 10, and 12), (b) a two-factor model with the dietary and PA subscales of the PDAP as latent variables, and (c) a one-factor model with PSE as latent variable. The last two models were tested as rival alternatives to the context-based model. Models, model modifications, and model fit are displayed in Table 2. The context-based, four-factor model fitted the data, as suggested by the model fit indices [33]. However, model fit was not better than acceptable, but better than for alternative models.

**Table 2:** Models, Model Modifications, and Model Fit for Confirmatory Factor Analyses with Maximum Likelihood Robust Estimation on Parental Self-Efficacy Items (*n* = 349)

Model	Model modifications	Model fit index			
		RMSEA (90 % CI)	CFI	SRMR	PGFI
Four-factor model^a^	Items 1–2, 15–17, 18–20, 23–24	0.09 (0.08, 0.09)	0.90	0.08	0.67
Two-factor model^b^	Items 1–2, 14–15, 18–20, 19–21	0.13 (0.12, 0.13)	0.74	0.09	0.54
One-factor model^c^	Items 1–2, 3–4, 14–15, 18–20	0.14 (0.13, 0.14)	0.70	0.10	0.54

Items 6, 10, and 12 were excluded from the analyses. Model modifications were conducted by correlating error terms of items. CI = confidence interval, RMSEA = root mean square error of approximation, CFI = comparative fit index, SRMR = standardized root mean square residual, PGFI = parsimony goodness-of-fit index

^a^Four-factor model: latent variables obtained using exploratory factor analysis

^b^Two-factor model: the dietary and physical activity subscales of the parental self-efficacy instrument as latent variables

^c^One-factor model: parental self-efficacy as latent variable

### Correlations between parental self-efficacy and parent-reported child behaviors

Correlations between PSE and parent-reported dietary, PA, and sedentary behaviors in children were all in the expected direction: positive correlations between PSE and healthy behaviors, and negative correlations between PSE and unhealthy behaviors. Correlations were in the low range (all *r*_s_s ≤ .32). In general, the strength of correlation varied depending on whether the PDAP factors represented dietary or PA behaviors: Factors 1 and 2 were more strongly correlated with dietary behaviors, whereas Factors 3 and 4 were more strongly correlated with PA and sedentary behaviors. For correlations between PSE and dietary, PA, and sedentary behaviors in children, see Table 3.

**Table 3:** Associations between Parental Self-Efficacy and Parent-Reported Dietary and Physical Activity Behaviors in Children (*n* = 698)

Dietary and physical activity behaviors	Parental self-efficacy				
	Total scale	Factor 1^a^	Factor 2	Factor 3	Factor 4
Vegetables	.22**	.20**	.19**	.15**	.19**
Fruits	.19**	.13**	.13**	.14**	.17**
White fish (e.g., cod)	.14**	.16**	.08*	.10**	.10**
Oily fish (e.g., salmon)	.16**	.17**	.10**	.13**	.15**
Fruit juice	.00	-.04	-.06	.02	.04
Fruit drink (w/ sugar)	-.03	-.02	-.08*	-.02	-.02
Fruit drink (w/o sugar)	.00	-.02	-.02	.04	.02
Soft drink (w/ sugar)	-.09*	-.12**	-.11**	-.01	-.06
Soft drink (w/o sugar)	-.03	-.08*	-.06	.00	.01
Chocolate drink	-.03	-.04	-.08*	-.03	.02
Milk (0,5 % fat)	.09*	.00	.11**	.07	.11**
Milk (1,5 % fat)	-.04	-.02	-.02	-.01	-.05
Milk (3 % fat)	-.01	.02	-.03	-.04	-.01
Ice cream	-.09*	-.05	-.16**	-.01	-.06
Candy	-.09*	-.06	-.17**	-.03	-.07
Cake/cookies/buns	-.13**	-.14**	-.11*	-.10*	-.08*
Chips/popcorn/cheese doodles	-.09*	-.11**	-.17**	-.02	-.03
Hamburger/pizza/Doner kebab	-.19**	-.19**	-.17**	-.12**	-.13**
French fries	-.17**	-.18**	-.15**	-.14**	-.09*
Play outside (weekdays)	.21**	.11**	.10*	.22**	.21**
Play outside (weekends)	.25**	.06	.11**	.32**	.29**
Play electronic games (weekdays)	-.16**	-.15**	-.17**	-.10**	-.13**
Play electronic games (weekends)	-.18**	-.12**	-.13**	-.17**	-.17**
Watch shows or movies (weekdays)	-.12**	-.06	-.10**	-.13**	-.10**
Watch shows or movies (weekends)	-.21**	-.11**	-.15**	-.20**	-.18**
Organized leisure time activity (Tuesdays)^b^	.16	.19*	.10	.12	.15
Organized leisure time activity (Saturdays)^c^	.14	.04	.06	.22**	.16

All correlations are Spearman

^a^Factor labels. Contextual circumstances that 1) Facilitate parental self-efficacy (PSE) for promoting healthy dietary behaviors in children; 2) Impede PSE for promoting healthy dietary behaviors in children; 3) Facilitate PSE for promoting healthy PA behaviors in children; and 4) Impede PSE for promoting healthy PA behaviors in children

^b^ *n* = 141

^c^ *n* = 151

*p < .05

**p < .01

## Discussion

In the present study, a new instrument of context-based PSE, the PDAP, was developed and evaluated psychometrically. In contrast to previous PSE measures, the PDAP items were designed exclusively to reflect contextual factors that either facilitate or impede PSE for promoting healthy dietary and PA behaviors in children. Interviews showed that participants perceived the PDAP items as highly comprehensible, relevant and acceptable. In a CFA of a 21-item version of the PDAP (excluding three items), a four-factor model differentiating between contextual circumstances fitted the data, suggesting that context is pertinent to the construct of PSE, as argued by SE theory [9]. Internal consistency was excellent for the total scale and good for its factors. The acceptable model fit lends preliminary support of construct validity. However, the model has to be empirically validated in a new sample before definitive conclusions can be drawn about construct validity. Reasons for the moderate model fit may be that following EFA items were excluded based on theory only, not to optimize factor solution, and that only the theory-based four-factor structure was examined using EFA, rather than testing a number of different factor solutions. Factor analysis should be based on theory [33], rather than aiming at optimizing factor solution or model fit for its own sake.

Three items were problematic and excluded from the EFA factor solution and the subsequent CFA. Item 6 loaded on an unexpected factor, for unclear reasons. An explanation might be that PSE in the domains of dietary and PA behaviors in children tend to overlap (as suggested by the high factor correlations and the relatively low factor loadings). Possible reasons for this overlap are the interrelatedness of efficacy beliefs in proximate domains (i.e., between-domain generality; [9]), and the clustering of child behaviors in these domains (e.g., [13]). Loadings for items 10 and 12 were low and concerned several factors. These two items assess PSE in contexts rather different from the contexts of the other items, for example, parties and holidays. On these special or rare occasions, engaging in unhealthy dietary behaviors may be an integral part in many families, and parents may not perceive these contexts as relevant for their efficacy beliefs. Thus, such items may not fit into a scale of contextual influences upon PSE. Therefore, items 10 and 12 should be considered for exclusion in future uses of the PDAP, together with item 6. In this context, items 23 and 24 deserve attention. These items were intended to represent contextual factors impeding PSE for promoting healthy PA behaviors in children (Factor 4). Interestingly, however, these items were considered by participants as constituting contextual circumstances *facilitating* PSE for these behaviors (Factor 3).

Correlations between PSE and parent-reported child behaviors were in the expected direction, which depended on whether behaviors were healthy or unhealthy, adding preliminary support of construct validity of the PDAP. In general, correlations were stronger when factor content and behaviors covered the same domain (e.g., PA). However, as in previous studies (e.g.,[23, 24, 30]), correlations were in the low range. One reason may be that the PDAP concerns PSE for *parental* behaviors promoting healthy child behaviors, and not *child* behaviors per se. It is possible that correlations would have been larger if PSE had been correlated with parental behaviors. For example, it is reasonable to expect that correlations between PSE for promoting physical play and parental behaviors aiming at promoting physical play (e.g., talking positively about playgrounds, purchasing equipment for physical play) would be larger than correlations between PSE for promoting physical play and children’s physical play. Another reason may be that in addition to PSE a host of other environmental as well as genetic factors influence these behaviors in children [35].

There are several limitations of the present study. The response rate was low and participants were mothers only of whom the majority was well-educated. However, when comparing PSE of participants who had not studied at a higher education institution (*n* = 129) with those that had (*n* = 569), levels were similar (former group means, total scale = 177, Factor 1 = 40, Factor 2 = 35, Factor 3 = 58, and Factor 4 = 44; latter group means, total scale = 181, Factor 1 = 42, Factor 2 = 36, Factor 3 = 59, and Factor 4 = 44). Also, there were differences between participants and non-participants on marital status and ethnicity. These circumstances may constrain the generalizability of the results. Other limitations concern the assessment of child behaviors, which were parent-reported. The items on PA and sedentary behaviors were developed as part of the study and their psychometric properties are not known. Assessing PA behaviors in children objectively using accelerometry is the preferred method [36], however, parent-report of dietary behaviors is required in populations of preschool children, and considered a valid and resource-saving method [37, 38].

## Conclusions

The PDAP was developed to assess PSE for promoting healthy dietary and PA behaviors in children under contextual circumstances either facilitating or impeding successful performance. Measuring PSE in relation to context rather than quantity or frequency may provide a more valid assessment of the construct, and give better indications of what behaviors to target and when in childhood obesity interventions. The present psychometric evaluation of a 21-item version of the PDAP provided preliminary support of construct validity. Confirmatory factor analysis yielded acceptable fit of a context-based model. Correlations between PSE and child behaviors were in the expected direction (i.e., positive correlations between PSE and healthy behaviors, negative correlations between PSE and unhealthy behaviors). Internal consistency of the PDAP and its factors was good to excellent.

## Abbreviations

BMI: Body mass index
CFA: Confirmatory factor analysis
CFI: Comparative fit index
EFA: Exploratory factor analysis
FFQ: Food frequency questionnaire
PA: Physical activity
PDAP: Parental self-efficacy for healthy dietary and physical activity behaviors in preschoolers scale
PGFI: Parsimony goodness-of-fit index
PSE: Parental self-efficacy
RMSEA: Root mean square error of approximation
SCT: Social cognitive theory
SRMR: Standardized root mean square residual

## References

[1] World Health Organization. Global Strategy on Diet, Physical Activity, and Health: Childhood Overweight and Obesity. 2015. http://www.who.int/dietphysicalactivity/childhood/en/. Accessed 8 Sept 2015.

[2] Daniels SR (2009). Complications of obesity in children and adolescents. Int J Obes (Lond).

[3] Griffiths LJ, Parsons TJ, Hill AJ (2010). Self-esteem and quality of life in obese children and adolescents: a systematic review. Int J Pediatr Obes.

[4] Singh AS, Mulder C, Twisk JW, van Mechelen W, Chinapaw MJ (2008). Tracking of childhood overweight into adulthood: a systematic review of the literature. Obes Rev.

[5] Reilly JJ, Kelly J (2011). Long-term impact of overweight and obesity in childhood and adolescence on morbidity and premature mortality in adulthood: systematic review. Int J Obes (Lond).

[6] Oude Luttikhuis H, Baur L, Jansen H, Shrewsbury VA, O'Malley C, Stolk RP et al. Interventions for treating obesity in children. Cochrane Db Syst Rev. 2009(1). doi:Artn Cd001872Doi 10.1002/14651858.Cd001872.Pub2. 10.1002/14651858.CD001872.pub2 19160202

[7] Waters E, de Silva-Sanigorski A, Hall BJ, Brown T, Campbell KJ, Gao Y (2011). Interventions for preventing obesity in children. Cochrane Database Syst Rev.

[8] Nixon CA, Moore HJ, Douthwaite W, Gibson EL, Vogele C, Kreichauf S (2012). Identifying effective behavioural models and behaviour change strategies underpinning preschool- and school-based obesity prevention interventions aimed at 4-6-year-olds: a systematic review. Obes Rev.

[9] Bandura A (1997). Self-efficacy: the exercise of control.

[10] Bandura A, Smith KG, Hitt MA (2005). The evolution of social cognitive theory. Great minds in management.

[11] Bandura A (2004). Health promotion by social cognitive means. Health Educ Behav.

[12] Bandura A (2012). On the functional properties of perceived self-efficacy revisited. J Manage.

[13] Gubbels JS, Kremers SP, Stafleu A, Goldbohm RA, de Vries NK, Thijs C (2012). Clustering of energy balance-related behaviors in 5-year-old children: lifestyle patterns and their longitudinal association with weight status development in early childhood. Int J Behav Nutr Phys Act.

[14] Rhee K (2008). Childhood overweight and the relationship between parent behaviors, parenting style, and family functioning. Ann Am Acad Polit Ss.

[15] Bandura A, Pajares F, Urdan T (2006). A guide for constructing self-efficacy scales. Self-efficacy beliefs of adolescents.

[16] Maibach E, Murphy DA (1995). Self-efficacy in health promotion research and practice: conceptualization and measurement. Health Educ Res.

[17] Pajares F, Hartley J, Valiante G (2001). Response format in writing self-efficacy assessment: greater discrimination increases prediction. Meas Eval Couns Dev.

[18] Price SM, McDivitt J, Weber D, Wolff LS, Massett HA, Fulton JE (2008). Correlates of weight-bearing physical activity among adolescent girls: results from a national survey of girls and their parents. J Phys Act Health.

[19] Hildebrand DA, Betts NM (2009). Assessment of stage of change, decisional balance, self-efficacy, and use of processes of change of low-income parents for increasing servings of fruits and vegetables to preschool-aged children. J Nutr Educ Behav.

[20] Taveras EM, Mitchell K, Gortmaker SL (2009). Parental confidence in making overweight-related behavior changes. Pediatrics.

[21] Adkins S, Sherwood NE, Story M, Davis M (2004). Physical activity among African-American girls: the role of parents and the home environment. Obes Res.

[22] Weber Cullen K, Baranowski T, Rittenberry L, Cosart C, Owens E, Hebert D (2000). Socioenvironmental influences on children's fruit, juice and vegetable consumption as reported by parents: reliability and validity of measures. Public Health Nutr.

[23] Wright JA, Adams WG, Laforge RG, Berry D, Friedman RH (2014). Assessing parental self-efficacy for obesity prevention related behaviors. Int J Behav Nutr Phys Act.

[24] Campbell K, Hesketh K, Silverii A, Abbott G (2010). Maternal self-efficacy regarding children's eating and sedentary behaviours in the early years: associations with children's food intake and sedentary behaviours. Int J Pediatr Obes.

[25] Decker JW (2012). Initial development and testing of a questionnaire of parental self-efficacy for enacting healthy lifestyles in their children. J Spec Pediatr Nurs.

[26] Smith BJ, Grunseit A, Hardy LL, King L, Wolfenden L, Milat A (2010). Parental influences on child physical activity and screen viewing time: a population based study. BMC Public Health.

[27] Fiese BH, Team SK (2013). Context matters in pediatric obesity: commentary on innovative treatment and prevention programs for pediatric overweight and obesity. J Pediatr Psychol.

[28] Caprara GV, Regalia C, Scabini E, Barbaranelli C, Bandura A (2004). Assessment of filial, parental, marital, and collective family efficacy beliefs. Eur J Psychol Assess.

[29] Bohman B, Ghaderi A, Rasmussen F. Psychometric properties of a new measure of parental self-efficacy for promoting physical activity and dietary behaviors in children. Eur J Psychol Assess. 2013;29(4):291–98

[30] Bohman B, Nyberg G, Sundblom E, Schafer EL (2013). Validity and reliability of a parental self-efficacy instrument in the Healthy School Start prevention trial of childhood obesity. Health Educ Behav.

[31] Doring N, Hansson LM, Andersson ES, Bohman B, Westin M, Magnusson M et al. Primary prevention of childhood obesity through counselling sessions at Swedish child health centres: design, methods and baseline sample characteristics of the PRIMROSE cluster-randomised trial. BMC Public Health. 2014;14. doi:Artn 335Doi 10.1186/1471-2458-14-335. 10.1186/1471-2458-14-335 PMC3995501 24717011

[32] Jöreskog KG (2003). Factor analysis by MINRES.

[33] Tabachnick BG, Fidell LS (2007). Using multivariate statistics.

[34] Kline RB (2005). Principles and practice of structural equation modeling.

[35] Harrison K, Bost KK, McBride BA, Donovan SM, Grigsby-Toussaint DS, Kim J (2011). Toward a developmental conceptualization of contributors to overweight and obesity in childhood: the Six-Cs Model. Child Dev Perspect.

[36] Adamo KB, Prince SA, Tricco AC, Connor-Gorber S, Tremblay M (2009). A comparison of indirect versus direct measures for assessing physical activity in the pediatric population: a systematic review. Int J Pediatr Obes.

[37] Bell LK, Golley RK, Magarey AM (2013). Short tools to assess young children's dietary intake: a systematic review focusing on application to dietary index research. J Obes.

[38] Bennett CA, de Silva-Sanigorski AM, Nichols M, Bell AC, Swinburn BA (2009). Assessing the intake of obesity-related foods and beverages in young children: comparison of a simple population survey with 24 h-recall. Int J Behav Nutr Phys Act.

